# Acupuncture for vertebrobasilar insufficiency vertigo: Protocol for a systematic review and meta-analysis

**DOI:** 10.1097/MD.0000000000009261

**Published:** 2017-12-15

**Authors:** Xiaohui Li, Menghui Liu, Yu Zhang, Ziqing Li, Dawei Wang, Xia Yan

**Affiliations:** a Guangzhou University of Chinese Medicine; b The First Affiliated Hospital, Sun Yet-sen University; c The Second Affiliated Hospital of Guangzhou University of Chinese Medicine, Guangdong Provincial Hospital of Chinese Medicine, Guangzhou, China.

**Keywords:** acupuncture, protocol, systematic review, vertebrobasilar insufficiency vertigo

## Abstract

**Background:**

Vertebrobasilar insufficiency vertebrobasilar (VBIV) is a serious nerve disease and many old people suffer from it. Acupuncture has been widely applied in the treatment of VBIV. However, to our knowledge, there has been no systematic review or meta-analysis of randomized controlled trails regarding the effectiveness of this treatment. Here, we provide a protocol to evaluate the effectiveness and safety of acupuncture for VBIV.

**Methods:**

Relevant randomized controlled trials in 5 databases (EMBASE, PubMed, the Cochrane Central Register of Controlled Trials [Cochrane Library], Chinese Biomedical Literature Database [CBM], China National Knowledge Infrastructure [CNKI]) will be comprehensively searched by 2 researchers in December 2017. The clinical efficacy will be accepted as the primary outcomes. We will also use RevMan V.5.3 software to compute the data synthesis carefully when a meta-analysis is allowed.

**Results:**

This study will provide a high-quality synthesis of current evidence of acupuncture for VBIV from several aspects including clinical efficacy, the blood flow velocity of the left vertebral artery (LVA), the right vertebral artery (RVA), the basilar artery (BA)and adverse events.

**Conclusion:**

The conclusion of our systematic review will provide evidence to judge whether acupuncture is an effective and safety intervention for patient with VBIV.

## Abstract 2

Supplemental Digital Content is available in the text

## 1 Introduction

Vertebrobasilar insufficiency (VBI) is defined as transitory ischemia of the vertebrobasilar circulation.^[1]^ It is induced by head extension or rotation that impairs the flow through the basilar arteries or vertebral, leading to ischaemia of the cerebellum, brain-stem, occipital lobes, and thalamus.^[2]^ For the reason that VBI is a clinical syndrome, it can contribute significantly to vertigo or dizziness, eye rotation, sensorineural hearing loss, syncope, and brain, or cerebral stem ischaemia, leading to declining in quality of life and even death.^[3]^ Especially, Vertigo associated with VBI is frequently associated with other neurological disturbances which bring huge pain and inconvenience to the patient's life and work.^[4]^

Up to now, first-line treatment for VBIV includes medical therapy (such as antithrombotic agents) and operative treatment (such as stenting, extracranial and intracranial, and surgical revascularization).^[5]^ However, both systemic and local side effects of them have been reported, for example, traditionally prescribing antithrombotic agents too often increases the risk of bleeding^[6]^ and may cause the operating plaque rupture, which can lead to complications, for instance, thrombosis and arterial vasospasm.^[7]^

Fortunately, complementary and alternative medicine, such as acupuncture, a form of therapy which stimulates acupoints by inserting filiform needles, can alleviate the symptoms of dizziness effectively and have very few side effects.^[8,9]^Currently, there are plenty of clinicians in China and a lot of Western countries that strongly recommend acupuncture treatment for patient with vertigo.^[10]^ A large numbers of clinical studies^[11–13]^ have shown that acupuncture may have promising alternative therapeutic effectiveness for VBIV. According to research, acupuncture has a positive effect on the release of tissue around the neck, regulating the flow of Qi and blood, nourishing the brain to improve blood supply to the brain;^[14]^ for instance, Fengchi (GB 20), Lieque (LU 7), and Baihui (GV 20), were thought to improve blood supply to the vertebral arteries and accelerate velocity of blood flow.^[15,16]^ Although its exact mechanism is still unknown, The National Institutes of Health (NIH) recommended that acupuncture could be used as an adjunct treatment in many diseases including VBIV.^[17]^

However, there are still some studies that produce inconsistent or contradictory findings, possibly due to the limitations of individual studies.^[18]^ Therefore, we will perform a meta-analysis of published studies to shed light on these contradictory results. According to current knowledge, there has been no systematic evaluation and meta-analysis of the acupuncture treatment for VBIV. Hence, we will conduct a comprehensive and quantitative evaluation analysis to assess its effectiveness and safety in clinical treatment. This study will be following the recommendations of the Cochrane Collaboration.

## 2 Methods

### 2.1 Inclusion criteria for study selection

#### 2.1.1 Types of studies

Randomized controlled trials (RCTs) of acupuncture for patients with the VBIV will be included without restrictions on language and publication status.

#### 2.1.2 Types of patients

Patients with the VBIV regardless of age, gender, educational status, sex, and stage will be included.

#### 2.1.3 Types of interventions

Acupuncture treatment includes various types of stimulation (such as manual, electroacupuncture, fire needle, warm acupuncture, and scalp acupuncture). Any methods can be used without limitations on the treatment length and frequency. The control groups without intervention, placebo control, sham acupuncture, and drug injection therapy will be included. The combined therapy can be accessed in the acupuncture group if the combined therapy has no difference in both groups. To evaluate the efficacy of acupuncture accurately, we plan to compare acupuncture treatment with either no interaction or sham acupuncture.

#### 2.1.4 Types of outcome measures

##### 2.1.4.1 Primary outcomes

The primary outcome will be defined as clinical efficacy. It will be assessed based on the following 3 categories. The indication of recovery includes completed disappearance of clinical symptoms, the improvement of signs, and integrated normal of physical index of the transcranial Doppler (TCD) examination of the blood flow in the vertebrobasilar arteries. Validity shows that not only the clinical symptoms will alleviate, but also the signs improve, and that the physical index of the TCD examination of the blood flow in the vertebrobasilar arteries will be efficiently improved. Invalidity indicates that the clinical symptoms, the main signs, and the physical index of TCD examination of vertebrobasilar arteries blood will continue without any change.

##### 2.1.4.2 Secondary outcomes

The secondary outcome will be the velocity of the blood flow in the vertebrobasilar artery by TCD, comprising the blood flow velocity of the left vertebral artery (LVA), the right vertebral artery (RVA), and the basilar artery (BA). In addition, side effects will also be considered.

### 2.2 Search methods for the identification of studies

The following 5 databases regardless of the publication status will be searched electronically EMBASE, PubMed, the Cochrane Central Register of Controlled Trials (Cochrane Library)), Chinese Biomedical Literature Database (CBM), China National Knowledge Infrastructure (CNKI) from inception to December 2017. The strategy will be created according to the Cochrane handbook guidelines. Search terms will be as follows: acupuncture, vertebrobasilar insufficiency vertigo, and randomized controlled trials. The search strategy for PubMed will be shown in Appendix 1.

#### 2.2.1 Searching other resources

We will search potential eligible studies through relevant conference proceedings and reference list of anteriorly published reviews.

### 2.3 Data collection and analysis

#### 2.3.1 Selection of studies

**Figure 1:**
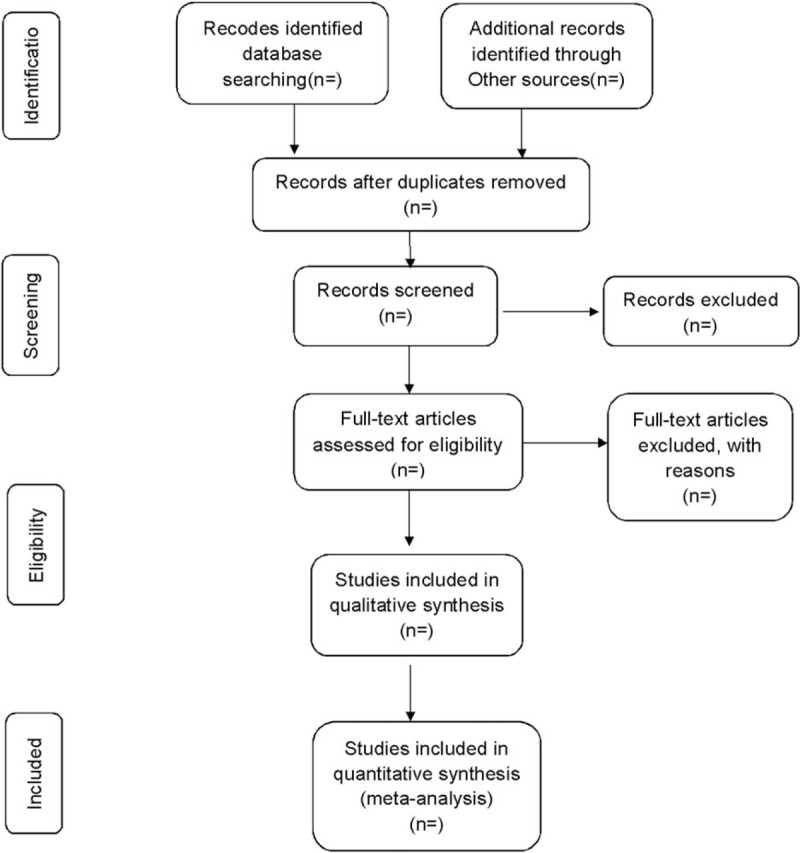
Flow diagram of study selection process.

All reviewers will receive training to ensure a basic understanding of the purpose and process of the review. The studies searched from electronic searching and other sources will be moved to a database set up by Endnote X7. Two reviewers will independently screen the titles, abstracts and keywords of all the potentially eligible references and decide which trials can satisfy the inclusion criteria (Fig. 1). Any divarication will be resolved through discussion between 2 reviewers to reach consensus. We will consult a third reviewer for arbitration when necessary.

#### 2.3.2 Data extraction and management

Data from the selected reports or studies will be extracted and filled in the data extraction form by 2 authors independently. We will extract information for reference ID, author, time of publication, characteristics of participants, blinding, interventions, and other information. We will be in contact with authors of trials for further information when necessary.

#### 2.3.3 Assessment of risk of bias in included studies

The risk of bias will be evaluated by 2 reviewers based on the Cochrane collaboration's tool.^[19]^ We will assess the following 7 domains: random sequence generation, allocation concealment, blinding of participants, personnel and outcome, incomplete outcome data addressed, selective reporting, and other bias. The risk of bias will be classified as low, unclear, and high.

#### 2.3.4 Dealing with missing data

We will attempt to correspond with the original investigator of the included studies to retrieve any missing or inadequate trial data. If the missing data cannot be obtained, we will select only the available data to decrease the potential influence of the missing data.

#### 2.3.5 Data synthesis and analysis

We will use RevMan V.5.3 software from the Cochrane Collaboration to perform data synthesis when a meta-analysis is feasible. Standard mean difference (SMD) with 95% confidence interval (CI) will be applied to evaluate continuous outcomes, while dichotomous outcomes will be analyzed by the relative risk (RR) with 95% CIs. We will perform *I*^2^ statistic to quantify inconsistencies among the included trials.

If the heterogeneity test shows little heterogeneity (*I*^2^ < 50%), the fixed effect model will be used for pooled data. If the heterogeneity is large (*I*^2^ ≥ 50%), the random effect model will be used for pooled data, and we will perform a subgroup analysis for these factors, such as the type of acupuncture and acupoint, are likely to lead to outcome bias. In addition, the sensitivity analysis will also be employed for exploring the causes of heterogeneity. If meta-analysis is not applicable, narrative synthesis will be used.

#### 2.3.6 Assessment of reporting bias

When the trials included in a meta-analysis are more than 9, funnel plots will be generated to detect the reporting bias. We will perform Egger's test to assess plots visually.

#### 2.3.7 Subgroup analysis

If we identify substantial heterogeneity, subgroups analysis will be performed depending on different interventions, controls and outcome measures.

#### 2.3.8 Sensitivity analysis

When sufficient trials are available, we will perform sensitivity analysis to identify whether the conclusions are robust in the review according to the following: (a) sample size; (b) the effect of missing data; and (c) methodological quality.

#### 2.3.9 Grading the quality of evidence

The Grading of Recommendations Assessment, Development and Evaluation (GRADE)^[20]^ will be performed to judge the quality of evidence in the main outcome. The quality of evidence will be assessed based on the following 4 levels: high, moderate, low, or very low.

## 3 Discussion

VBIV belongs to the categories of vertigo and dizziness in traditional Chinese medicine.^[21]^ Acupuncture have been cited for treating vertigo in many centuries,^[22]^ and the results of clinical studies^[23,24]^ suggest that acupuncture could demonstrate a significant and immediate effect on reducing discomforts of both dizziness and vertigo. However, up to now, there has been no overall assessment of the clinical evidence regarding acupuncture interventions for VBIV based on evidence-based medicine. Thus, we plan to conduct this systematic review and meta-analysis to estimate the efficacy and safety of acupuncture therapies for patients with VBIV. We expect this review will provide more convincing evidence to assist patients and clinicians to make right decision when dealing with VBIV. However, there are still some potential limitations of the proposed systematic review. First, there may be a bias on language since only the studies published in English and Chinese will be included. Hence, some relevant studies published in other languages (such as Japanese and Korean) might be missed. Second, a limitation of this review is that there may cause high heterogeneity because of several different types of acupoint, duration and frequency. Finally, due to the difficulty in taking blind measures during acupuncture treatment, it may bias the outcome.

PRISMA-P (Preferred Reporting Items for Systematic review and Meta-Analysis Protocols) checklist of this protocol is presented in online supplementary.

## Supplementary Material

Supplemental Digital Content
