# Supplementary material for: Acupuncture for vertebrobasilar insufficiency vertigo: Protocol for a systematic review and meta-analysis
Source: Medicine (Baltimore). 2017 Dec 15;96(50):e9261. doi: 10.1097/MD.0000000000009261 (PMC5815781; doi:10.1097/MD.0000000000009261)
Supplement: Supplemental Digital Content [file medi-96-e9261-s001.docx]

**Appendix 1.**

***Search strategy used in PubMed database***

#1 Insufficiencies, Vertebrobasilar OR Insufficiency, Vertebrobasilar OR Vertebrobasilar Insufficiencies OR Vertebro-Basilar Ischemia OR Ischemia, Vertebro-Basilar OR Ischemias, Vertebro-Basilar OR Vertebro Basilar Ischemia OR Vertebro-Basilar Ischemias OR Vertebrobasilar Ischemia OR Ischemia, Vertebrobasilar OR Ischemias, Vertebrobasilar OR Vertebrobasilar Ischemias OR Vertebro-Basilar Insufficiency OR Insufficiencies, Vertebro-Basilar OR Insufficiency, Vertebro-Basilar OR Vertebro Basilar Insufficiency OR Vertebro-Basilar Insufficiencies OR Vertebrobasilar Dolichoectasia OR Dolichoectasia, Vertebrobasilar OR Dolichoectasias, Vertebrobasilar OR Vertebrobasilar Dolichoectasias OR Vertebral Artery Insufficiency OR Artery Insufficiencies, Vertebral OR Artery Insufficiency, Vertebral OR Insufficiencies, Vertebral Artery OR Insufficiency, Vertebral Artery OR Vertebral Artery Insufficiencies OR Vertebral Artery Ischemia OR Artery Ischemia, Vertebral OR Artery Ischemias, Vertebral OR Ischemia, Vertebral Artery OR Ischemias, Vertebral Artery OR Vertebral Artery Ischemias OR Vertebral Artery Stenosis OR Artery Stenoses, Vertebral OR Artery Stenosis, Vertebral OR Stenoses, Vertebral Artery OR Stenosis, Vertebral Artery OR Vertebral Artery Stenoses OR Basilar Artery Insufficiency OR Artery Insufficiencies, Basilar OR Artery Insufficiency, Basilar OR Basilar Artery Insufficiencies OR Insufficiencies, Basilar Artery OR Insufficiency, Basilar Artery OR Basilar Insufficiency OR Basilar Insufficiencies OR Insufficiencies, Basilar OR Insufficiency, Basilar OR Basilar Artery Ischemia OR Artery Ischemia, Basilar OR Artery Ischemias, Basilar OR Basilar Artery Ischemias OR Ischemia, Basilar Artery OR Ischemias, Basilar Artery OR Basilar Artery Stenosis OR Artery Stenoses, Basilar OR Artery Stenosis, Basilar OR Basilar Artery Stenoses OR Stenoses, Basilar Artery OR Stenosis, Basilar Artery

#2 Acupuncture OR Acupoints. OR Acupuncture therapy. OR manual acupuncture. OR electroacupuncture. OR electro-acupuncture. OR ear acupuncture. OR Auricular acupuncture. OR scalp acupuncture. OR plum blossom needle. OR fire needling. OR dermal needle.

#3 Randomized controlled trial [Mesh] OR clinical study OR Clin-ical Trial OR Controlled study OR Controlled Trial OR Random*Control* study OR random* Control* Trial

#1 AND #2 AND #3
